# Carbon Fiber Reinforced Polymer Composites Doped with Graphene Oxide in Light of Spectroscopic Studies

**DOI:** 10.3390/ma14081835

**Published:** 2021-04-07

**Authors:** Paulina Florek, Magdalena Król, Piotr Jeleń, Włodzimierz Mozgawa

**Affiliations:** Faculty of Materials Science and Ceramic, AGH University of Science and Technology, 30 Mickiewicza Av., 30-059 Krakow, Poland; paulina@agh.edu.pl (P.F.); pjelen@agh.edu.pl (P.J.); mozgawa@agh.edu.pl (W.M.)

**Keywords:** laminate, CFRP–carbon fiber reinforced polymers, graphene oxide, DRIFT spectroscopy, Raman spectroscopy

## Abstract

Carbon fiber reinforced polymer composites are a dynamically developing group of lightweight composites for applications in the automotive, wind energy, aerospace, and sports sectors. Interfacial connection is the weakest place in these materials. In this study, an attempt was made to improve adhesion between carbon fiber and epoxy resin. For this purpose, nanoparticles of graphene oxide were added to a polymer matrix. The results of the three-point bend test showed that the strength of samples with added graphene oxide increased. Improvement of adhesion between components, reduction of the pull-out effect and change in the method of crack propagation were observed. An attempt was made to explain this effect using spectroscopic methods, both IR and Raman. On the basis of the obtained results, chemical bonds between the individual components of the composites were identified.

## 1. Introduction

Carbon fiber reinforced polymers (CFRP) have been used since the 1960s. Initially, this lightweight technology was extremely expensive and only affordable for aerospace and military industries [1]. As time passed, CFRP became more common [2,3]. Nowadays, this technology is used in a wide range of applications, such as in aerospace, automotive, and sports equipment. High strength to weight ratio, excellent corrosion resistance, and ease of handling makes CFRP competitive with materials such as steel, aluminum, concrete, or wood [4].

The main substrate of CFRP is carbon fibers (CFs). CFs are manufactured by the controlled pyrolysis of appropriate organic-based precursor fibers and consist of 92–95 wt.% carbon [5]. The specific structure of CFs gives them remarkably high tensile strength. On the other hand, their poorly defected surface has almost no functional groups, is nonpolar and non-reactive, and its wettability is insufficient. In order to increase the functionality of CFs as a reinforcement in CFRP, functional groups such as hydroxyl –OH, carboxyl –COOH, and carbonyl –CO [6,7,8] are introduced on their surface.

Composites are built of two basic phases. Reinforcement dispersed in a matrix is the stronger phase and mostly determines the strength of the composite. The matrix, as a continuous and weaker phase, is responsible for keeping the shape of the whole material and transferring the load on the reinforcement [4]. Additionally, taking into account that the matrix has contact with the environment, it determines the composite’s thermal, heat, and chemical resistance. Properly matched reinforcement and matrix guarantees good adhesion between the components, proper transfer of load, and that the highest possible mechanical parameters of the composite will be reached. Generally, composite manufacturers aim to create a product that will transfer as much load as possible, while maintaining the lowest possible weight at the same time. The weakest element of CFRP is the interfacial interaction between resin and CF, which is mostly only physical interlocking. Introducing chemical bonds between the components could significantly increase the strength of the material. This solution would certainly widen the range of applications of CFRP.

Various attempts to improve adhesion between resin and CFs can be found in the literature. Among them are:

- oxidation of CF (electrochemical [9], chemical [10], thermal [11], etc.)–results in forming reactive functional groups on the surface of fibers;
- microwave curing of the composites (instead of typical heating) [12]–improves the wettability of fibers;
- coating the surface of the fibers with nanoparticles (such as carbon nanotubes [13,14], carbon black [15], or graphene oxide [16,17]) using chemical vapour deposition (CVD), electrophoretic deposition [18] or chemical grafting [14,19]–increases reactivity and the specific surface area of CF.

One of the first studies on CFRP doped with nanoparticles concerned carbon nanotubes (CNTs). Depending on the amount of nanofiller introduced, increased mechanical properties of the material were obtained, such as a 16–36% increase in tensile strength [20]. Over time, the research evolved and the introduction of modified CNTs with better-defined structures began [21,22,23,24,25]. More recently, Lavagna et al. doubled composite apparent strength by grafting CNTs on the surface of carbon microfibers [26]. Islam et al. drafted CNTs onto the CF surface with strong carbon-carbon covalent bonding and obtained the failure stress from in situ SEM pulling out experiments in the range of 25 to 31 GPa [27]. However, the use of CNT as an additive for increasing mechanical parameters has ceased to be attractive for economic reasons. With the development of nanotechnology, cheaper and easier to manufacture graphene derivatives with a less complex structure have appeared on the market. Currently, research on the introduction of CNTs into carbon-polymer composites focuses on modification of their thermal and electrical conductivity and the development of functional materials, whereas to improve the mechanical parameters of composites, technologically more profitable solutions are being investigated.

The most promising nano additives for reinforcing CFRP are graphene-related materials (GRM). The consequence of the nanometric size is a large specific surface area, very high reactivity and low structure defects. By bonding with carbon fiber and resin, they improve the mechanical properties of laminates by developing interfacial connections [28]. Doping the matrix with nanoparticles is an easy method to strengthen CFRP that does not require specialized equipment. Potts et al. [29] and Hu et al. [30] present an overview of the results of research on CFRP, which proves the positive influence of GRMs on mechanical properties. The work [28] presents tests showing a 25% increase in the strength of a composite with 0.25% graphene addition. The optimal addition of nanoparticles in each case did not exceed 1 wt.%. The greater amount of graphene causes a decrease in the strength of the samples, which is explained by the negative influence of nanoparticles on the chemical reactions between the hardener and the resin. A large addition of graphene is also problematic in obtaining a homogeneous structure [31].

Graphene oxide (GO) is also considered in research on the modification of CFRP with nanoparticles. The structure of GO is less perfect than that of graphene because its structure has defects in the form of open six-membered carbon rings. The consequence of an imperfect network is reduced GO mechanical properties. However, this is irrelevant when it is used as a reinforcement in polymer-carbon composites, while the numerous functional groups in the discussed case have a great advantage [30,31] in the form of carbonyl and carboxyl groups connected to the ring with an sp^2^ bond and epoxy and hydroxy groups bounded to surface by an sp^3^ bond. The presence of the mentioned functional groups increases the solubility of GO in polar solvents, which makes it possible to disperse it well in the suspensions. Epoxy groups (oxirane rings) open easily and willingly react with CFs [32]. Han et al. [31] emphasize the multitude of works on CFRPs matrix reinforcement with graphene oxide (GO) nanoparticles, noting at the same time that only a small amount concerns full-size composites.

Considering the performance improvement of CFRP by GRM, two main approaches of doping should be discussed. One of the techniques is fabrication of GRM-modified fibers. Nanoparticles are grafted directly onto surface of the reinforcement. This increases the fiber surface area [33] and consequently improves the wettability of the reinforcement [34]. Grafting GRM results in probably the most desirable radial orientation of nanoparticles and alleviates the problem of agglomeration in the nanoparticles [35]. Although this method requires process development and specialized research equipment such as CVD or an electrophoretic system, it significantly increases performance of CFRP composites. On the other hand, GRM doped-matrix CFRP are being investigated. Simplicity and compatibility with most popular industrial techniques are its biggest advantages [36]. However, researchers deal with problems such as the agglomerating of GRM, self-filtration issues and matrix viscosity limits [35].

The doping of the matrix is currently the subject of intensive research as a method of reinforcing laminates that could be used on an industrial scale. The aim of this study was to investigate the effect of graphene oxide nanofiller on the properties of a composite composed of epoxy resin and carbon fiber fabric. Particular attention was paid to analysis of the transition zone between carbon fiber and epoxy resin (CF/ER) as the weakest element of the composite. Vibration spectroscopy (both IR and Raman) was chosen as the main research method, as it is a method by which it is possible to study specific chemical bonds. We would like to emphasize that in this type of research, Raman imaging was attempted for the first time in order to explain the interactions in the contact zone of composite components. The aim of the analyzes was to provide additional data on the interaction of the carbon nanofiller with both ER and CF and thus the mechanisms for improving the functional properties of CFRP composites.

Oxygen functional groups of GO provide an ambient path for strong binding with the polymer matrix. Different ways of dispersing nanoparticles in resin were investigated. Suspending graphene oxide in solvents commonly used for this purpose, such as ethyl alcohol, methyl alcohol or tetrahydrofuran, was considered. However, this is an additional, perhaps redundant step. Solvents that are improperly and incompletely removed from the resin can significantly affect the curing process. Therefore, in this work, contrary to the methods used in the literature [32], an attempt to homogeneously distribute GO directly in resin was made.

## 2. Materials and Methods

### 2.1. Materials

CFRP composites were obtained using low heat-treated carbon fiber fabric (LHT, 3k, 200 g/m^2^, 2 × 2 twill, Rymatex Sp. z o.o. (Rymanów, Poland), epoxy resin (ER) LG 700 with hardener HG 353 (GRM Systems Ltd.) (Olomouc, Czech Republic), and graphene oxide powder (GO) (purity >99%, diameter 0.5–3 μm, layers number <3, thickness 0.55–1.2 nm, carbon content 50–65 wt.%, Carbon Nanotubes Plus (Madisonville, TX, USA)).

### 2.2. Samples and Preparation

Three epoxy resin (ER) mixtures were prepared with 0.5, 1.0 and 1.5 wt.% GO addition. Mixtures were sonicated for 20 min (ultrasonic power–2 × 160 W, frequency–40 kHz) and then stirred for another 10 min. This procedure resulted in a homogeneous colloid of GO nanoparticles in the resin. Then, the colloids were mixed with the hardener and left to harden. There was no negative influence of GO on the resin hardening process.

**Figure 1:**
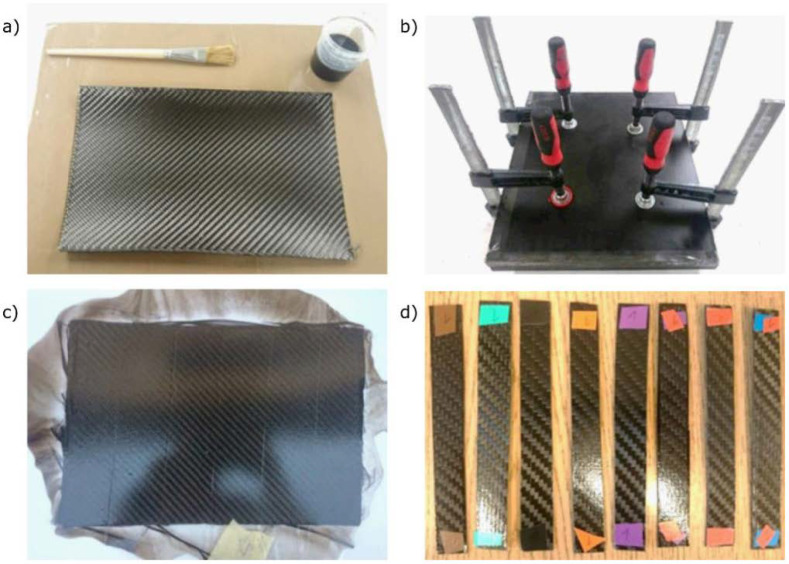
Stages of sample preparation: (**a**) preparation for the lamination process, (**b**) hardening of the laminate under pressure, (**c**) hardened composite, and (**d**) cut and marked samples for testing.

In the next step, CFRP with several types of matrix were prepared. Colloids differing in the amount of GO in the range of 0–1 wt.% were prepared as described above. For the manufacturing of each CFRP sample, four 200 × 300 mm pieces of carbon fiber fabrics were cut. The fabric layers were oriented at ±45° (as a result, the strength was evenly distributed; with this type of fabric weave and if there is no requirement that the laminate should be more durable in a given direction, this is the standard arrangement). The resin was evenly applied to the fabric with a brush. Diffusion of the resin throughout the carbon fiber fabric was achieved by applying pressure with a set of clamps. Samples were let to harden for 72 h and then taken out. Laminates were divided into rectangles with dimensions of 120 × 15 mm, cut out, and marked for further research. The steps of the sample preparation process are shown in Figure 1.

### 2.3. Instrumentation

Dynamic light scattering (DLS) measurements were made for epoxy resin samples doped with GO in order to verify dispersion of the filler in the matrix. DLS measurements were conducted on Zetasizer Nano-ZS from Malvern Instruments (Malvern, UK) (measurement temperature 25 °C, count rate 360.9 kcps, duration used 60 s).

The mechanical parameters of the samples were measured according to the EN ISO 14125:1998 standard for the three-point bending test. The test was carried out on a Zwick 1435 testing machine (ZwickRoell GmbH & Co., Ulm, Germany), coupled with deicated testXpert Master software version. Distance between supports was set to 63 mm and the crosshead speed to 1 mm/min. Every type of sample was measured 6 times.

The scanning electron microscopy (SEM) images of the CF and CFRP samples were obtained on a Nova NanoSEM 200 (FEI Company, Hillsboro, OR, USA) system. The surface of the composite was coated with gold, and an accelerating voltage of 18 kV was used during imaging.

Raman imaging was carried out on WITec Alpha 300 M+ apparatus (WITec Wissenschaftliche Instrumente und Technologie GmbH, Ulm, Germany). The spectrometer was equipped with 600 gr/mm grating and a 488 nm laser (laser power was set to prevent sample degradation). Zeiss Epiplan Neofluar 50× (Carl Zeiss AG, Oberkochen, Germany) long working objective was used, and laser spot was approx. 1 µm. The XY images were recorded on 30 × 30 µm spots with a 0.5 µm step, each lasting 1 s. The Z scan was performed at 40 × 10 µm in depth with 0.5 µm step and 1 s accumulations. Single spectrum measurements (graphene oxide) were collected with the same setup, and two 20 s scans were collected. Data processing was carried out using WITec Project Five 5.3 PRO software (WITec Wissenschaftliche Instrumente und Technologie GmbH, Ulm, Germany). All obtained spectra were subjected to despike filter and baseline correction.

The DRIFT spectra were obtained with a VERTEX 70v vacuum spectrometer (Bruker, Billerica, MA, USA) equipped with a Praying Mantis™ diffuse reflectance cell (Thermo Fisher Scientific, Waltham, MA, USA). Spectra were collected at room temperature in the range of 4000–400 cm^−1^ over 128 scans and at 4 cm^−1^ resolution. The Kubelka-Munk function was used to achieve a partial linearization [37].

## 3. Results and Discussions

### 3.1. Characterization of Starting Materials

**Figure 2:**
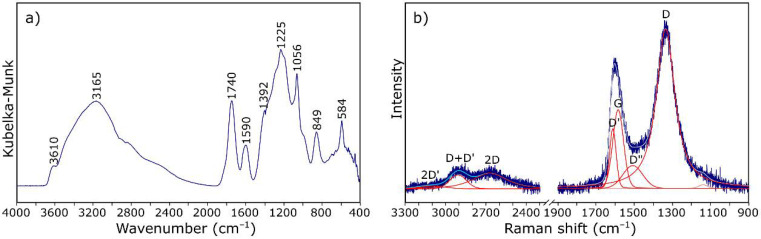
IR (**a**) and Raman spectrum (**b**) of graphene oxide (GO).

IR spectroscopy is one of the methods of structural research that can determine which functional groups are present in the analyzed material. Infrared spectroscopy allows for analysis of both the structure of molecules and their interaction with the environment. The functional groups present in the structure of GO were evaluated by DRIFT spectrum measurement, which was presented in Figure 2a. The spectrum envelope is in agreement with those reported in previous studies [38,39,40]. The broad band with the maximum at about 3165 cm^−1^ is attributed to stretching vibrations of hydroxyl –OH group. Bands at 1740, 1590, 1392, 1225, and 1056 cm^−1^ are characteristic for carbonyl C=O, aromatic C=C, carboxy C–O, epoxy C–O, and alkoxy C–O bonds, respectively.

Raman spectroscopy is an important technique for the structural characterization of carbon-based materials [41]. The Raman spectrum of crystalline graphite consists of a single, strong, first-order line at 1582 cm^−1^ (G band) due to the E_2g_ vibrational mode and second-order doublet at 2695 and 2735 cm^−1^ (G_1′_ And G_2′_ bands, respectively). For disordered carbons, additional bands appeared at 1360 cm^−1^ (D band), 1620 cm^−1^ (D′ band), and 2960 cm^−1^ (D″ band) [42,43,44,45].

Figure 2b shows the Raman spectrum of GO, in which the G-band occurs at about 1590 cm^−1^. It is characterized by a larger FWHM and a shift towards higher wavenumbers in relation to crystalline graphite [42]. The second band with higher integral intensity, at about 1335 cm^−1^, is the defect band (related to the oxidation of graphite). The appearance of both bands in the spectrum indicates the presence of graphite-like structures in the material. The group of bands occurring at lower frequencies (3000–2600 cm^−1^) indicates a greater order of the structure in relation to amorphous carbon. Taking into account the relative intensities of the D and G bands (ID/IG) and positions of the maxima of the 2D and D + D′ bands, it was found that the Csp2 percentage is about 50% [45].

**Figure 3:**
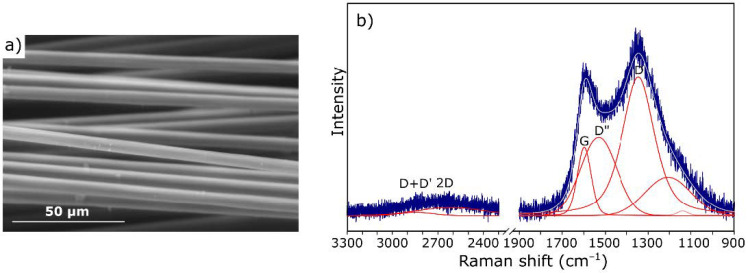
Scanning electron microscopy (SEM) images (**a**) and Raman spectrum (**b**) of the virgin carbon fibers (CFs).

For comparative purposes, samples of pure carbon fiber were also subjected to microscopic observations. An exemplary SEM image is shown in Figure 3a. Carbon fibers with a smooth surface without traces of sizing are visible. Such a microstructure affects the adhesion to the matrix and consequently the strength of the final composite.

The spectrum of carbon fiber (Figure 3b) also includes the D and G bands characteristic of carbon materials. Their shape confirms the fact that we are dealing with a fiber with a low degree of graphitization [46].

### 3.2. Characterization of ER Modified by GO

**Figure 4:**
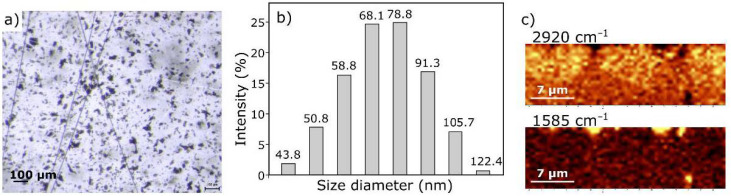
Microscopic photo (**a**), histogram of particle size distribution (**b**), and Raman images (the brighter the field, the greater the intensity) (**c**) of graphene oxide dispersed in epoxy resin.

GO is in the form of flakes with a size not exceeding a few micrometers (Figure 4a). Such forms tend to agglomerate. There are many methods for separating nanoparticles in the literature [47,48,49]. On the other hand, work on CFRP with graphene has shown [50] the significant impact of preparing nanoparticles on final composite properties. Comparing the results of tests on composites produced with the solvent method and the in-situ method reveals that the solvent method showed a better dispersion of graphene, while the problem was the solvent residues in the composite [50,51]. Therefore, as part of this study, an attempt was made to produce a composite with a GO-doped matrix without the use of a solvent.

**Figure 5:**
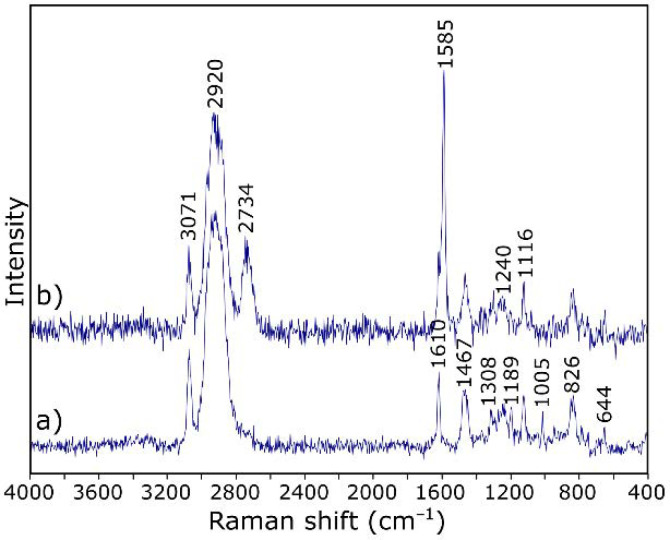
Raman spectra of (**a**) epoxy resin and (**b**) graphene oxide particle.

Figure 4 shows the images of GO dispersed in ER. The nanofiller particles evenly dispersed in the resin are visible in the microscopic photo (Figure 4a). A histogram showing the particle size distribution is shown in Figure 4b. Dispersion of the filler is satisfying as the range of the aggregates’ diameter is mostly between 58.77–91.28 nm. To locate GO within ER and confirm dispersion homogeneity, we also performed a Raman depth profile (Figure 4c). During calculations, normalized intensities of the bands at 2920 cm^−1^ (for resin) and 1585 cm^−1^ (for GO) were assumed. The choice of bands was dictated by the characteristic bands for the respective spectrum components (Figure 5). Bands at 2920 cm^−1^ are only due to C–H vibration and should be associated with the presence of resin, while the band at 1585 cm^−1^ is characteristic of GO (compare to Figure 2b). The color intensity in the Raman imagining (Figure 4b) is proportional to the intensity of the respective bands. It was confirmed that the visible inclusions with a size of approx. 1 µm have a graphene structure.

It is worth noting that the Raman spectrum of the starting GO (Figure 2b) has a slightly different course to the spectrum after dispersion in the resin (Figure 5b). After introduction to the resin, GO takes the so-called reduced form—the G band shifts towards lower wavenumbers (from about 1600 to 1585 cm^−1^) due to the increased number of sp^2^ carbon atoms [52]. The intensity of the D-band also decreases, indicating the disappearance of the oxygen-containing functional groups. Thus, it can be assumed that GO particles are linked to the resin by reactive functional groups, not only by hydrogen bonding, as suggested in the literature [36,53].

**Figure 6:**
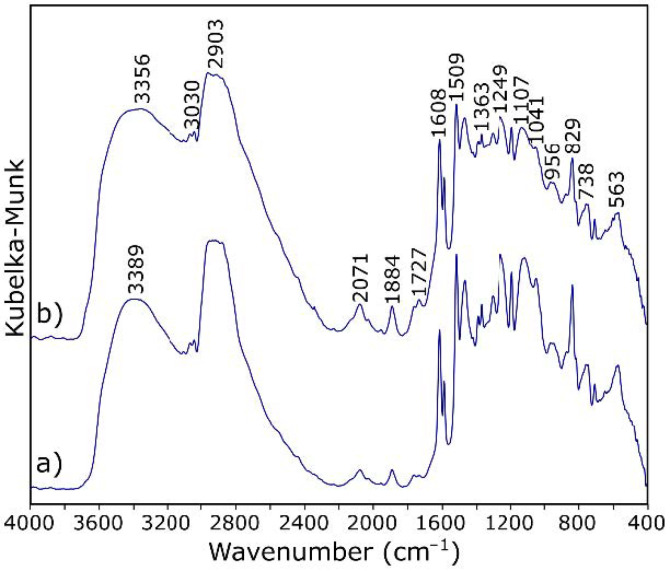
DRIFT spectra of (**a**) epoxy resin and (**b**) epoxy resin modified by graphene oxide.

Table 1 shows the assignation of bands for ER in the mid infrared range. In the DRIFT spectra (Figure 6a) of ER, the pattern of bands occurring in the range of 3000–2600 cm^−1^ is characteristic for epoxy resins. The broad vibrations band at about 3400 cm^−1^ arises from the O–H stretching modes.

In addition, the band at 3100 cm^−1^ is due to the vibration of the aromatic protons (aromatic –H stretch). The two absorption bands at about 2920 cm^−1^ and the band at 2867 cm^−1^ correspond to the symmetrical stretching vibrations of the methyl/methylene (alkyl) groups. The C–H bending vibrations of methyl are observed at 1383 and 1459 cm^−1^. The band with at about 1720 cm^−1^ arises from the carbonyl (C=O) stretch—its low integral intensity may indicate a good hardening of the resin. The characteristic C=C stretching of the benzene ring is observed at 1608, 1581, and 1509 cm^−1^. The bands observed at 1249 cm^−1^ are assigned to the vibrations of aromatic C–O in the epoxy ring. The characteristic absorption band of C–O–C for the epoxy group is observed at 915 cm^−1^—the very low intensity of this band shows again the high degree of cross-linking of the resin. Such a matrix will constitute a protective coating of the fibers, as well as to some extent participate in the transfer of loads to which the composite will be subjected.

The IR spectra of ER and ER modified by GO exhibit subtle changes (Figure 6). There are no differences in the ratios of the intensity and the FWHM of the individual bands. The background elevation >1700 cm^−1^ can be explained by the addition of graphene. Subtle changes are only observed in two ranges of wavenumbers. Compared with the reference sample (Figure 6a), in the spectrum of the material with added GO (Figure 6b), there is an additional maximum in the range of hydroxyl groups (at about 3356 cm^−1^). It can also be seen that the absorption band of carboxyl group (–C=O) in composites with GO (Figure 6b) appears at relatively low frequency (1727 cm^−1^) compared with its counterpart (1736 cm^−1^) when no GO was used. These observations are the basic characteristics of the occurrence of hydrogen bonding. The formation of hydrogen bonding strengthens the interaction between the ingredients in composites and thus reinforces the polymer matrix. Importantly [40], the hydroxyl and epoxy groups that can participate in the reaction with CF carbon fiber do not completely disappear.

### 3.3. Properties and Microstructure of CF/GO Composites

Table 2 shows the average values of the maximum transferred force **F_max_**, the maximum deflection **ε_max_**, bending stress **σ** and modulus of elasticity **E**.

The values of deformations **ε_max_**, bending stresses **σ**, and modulus of elasticity **E** show, to a certain extent, an upward trend with an increasing amount of nanofiller admixture. The results show that the optimal amount of GO in the matrix is 0.3 wt.%. The analyzed series of samples showed the greatest increase in mechanical properties compared to the reference sample (without GO). There was an increase of 18.4% and 35.5% for σ and E, respectively. It can be concluded that GO, introduced into the matrix in an appropriate amount, increases the ability of composites to deform, which results in the material’s ability to withstand higher stress.

After exceeding 0.3% of GO, the tested parameters begin to decrease gradually. The samples containing the highest amounts of nanofillers (0.5 and 1.0 wt.%) show mechanical properties at the level of the reference sample.

The obtained values of the optimal amount of GO additive correspond with the values obtained by other authors [30,31,56,57]. Han et al. [31] measured the strength of composites with 0.1 wt.% GO achieved an almost 10% increase in interlaminar shear strength. On the other hand, Pathak et al. [32] achieved a 25% increase in strength with 0.3% of the mass of GO. In turn, Shen et al. [56] observed that composites with the addition of 0.25 wt.% graphene showed 25% higher tensile strength than composites without graphene when the graphene content was more than 1% of the weight. Composites with graphene had worse properties than those made of undoped resins. Similar trends continued in the bending strength tests. In this case, only composites containing less than 0.5% of graphene showed 9% higher flexural strength than standard carbon-epoxy composites.

The slight discrepancies in the results obtained by different research groups arise from the use of different techniques for preparing composites. The method of the dispersion of nanoparticles in the matrix seems to be crucial (the suspension of graphene oxide in solvents was considered, such as ethyl alcohol, methyl alcohol, tetrahydrofuran or water. Suspensions were obtained by stirred and/or ultrasonication as well as wet mill). Researchers of the subject agree that agglomerates of nanoparticles should be as small as possible, as these may be a source of defects that reduce the strength of composites. They explain the increase in the strength parameters of the samples as a result of the formation of covalent bonds between epoxy resin and graphene oxide. In addition, graphene oxide is supposed to be a medium that facilitates the transfer of loads from the matrix to the reinforcement, acting as a kind of “bridge” as it chemically bonds with carbon fiber. GO improves interfacial interactions in the composite, thus preventing microcracks and delamination. The appropriate GO additive improves the mechanical properties of laminates, but at higher concentrations, GO tends to agglomerate, and inter-layer restacking has a detrimental effect on mechanical properties.

Another significant parameter characterizing composites is toughness, which is the amount of energy absorbed by the material during its destruction. One of the methods of its determination is calculating the area under the plot, **P_p_**, of stress, **σ**, as a function of strain **ε**. Table 1 shows the calculated average **P_p_** values for the tested samples. The amount of energy absorbed by the tested samples during destruction depends on the amount of added nanofiller. The changes are analogous to the modulus of elasticity or the transferred stresses, and they reach the optimal value, greater than the reference value by 18.8%, for samples with 0.3 wt.% GO content.

**Figure 7:**
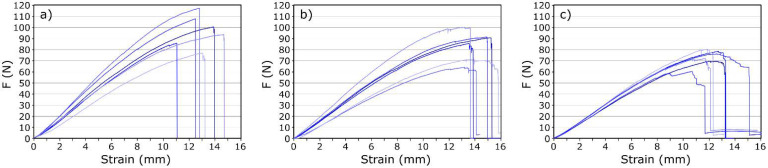
Stress-strain curves of three-point bending test samples for composites with: (**a**) 0; (**b**) 0.3, and (**c**) 0.5% graphene oxide (GO).

It is worth paying attention to the shape of the recorded stress-strain curves and the method of sample destruction. Examples of recorded curves are shown in Figure 7. One of the disadvantages of CFRP composites is their brittleness. Samples without the addition of nanoparticles break completely and suddenly, with no signals indicating the onset of deterioration (Figure 7a). GO improves the toughness of epoxy resin composites. The greater the mass fraction of GO, the more bends in the stress-strain curve, which result from gradual fracture of the composite. It can be concluded that the addition of GO changes the material deterioration from sudden to gradual.

**Figure 8:**
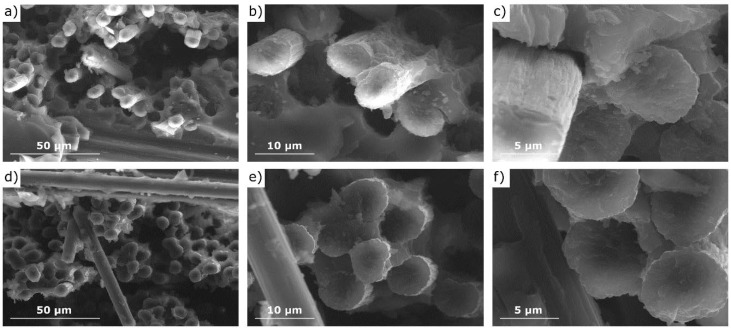
SEM images of the fractured surfaces of (**a**–**c**) the reference sample and (**d**–**f**) the sample with 0.3% GO addition.

To better understand the role of GO modification, the fracture surfaces of composites were investigated using SEM, as shown in Figure 8. In the case of the reference sample (Figure 8a–c), destruction occurred predominantly by progressive interfacial debonding and fiber pullout. In the place where the sample broke, the pulled fibers and the remaining holes are visible (Figure 8a,b). Microscopic observations confirm the lack of adhesion of the fibers and matrix. Gaps are observed at the interface between the fibers and the matrix (Figure 8c), which demonstrated the weak interfacial bonding between the matrix and fiber. In contrast, for the sample with the addition of nanofiller (Figure 8d–f), simultaneously broken fibers and the matrix can be observed. It can be concluded that the addition of GO promotes the adhesion of CFs and ER, which adheres to reinforcement, creating a continuous structure.

Similar observations related to inhibiting the pullout effect have been made by other authors [31]. It is well known that the weakest point of polymer-carbon laminates is the contact area of fiber and matrix [58]. The functional groups present on GO (C=C, epoxy C–O–C, hydroxyl C–OH, and carboxylic HO–C=O) can form interactions with both functional groups of CFs and ER. The interfacial bond created between the fiber and matrix increases the strength of composites.

**Figure 9:**
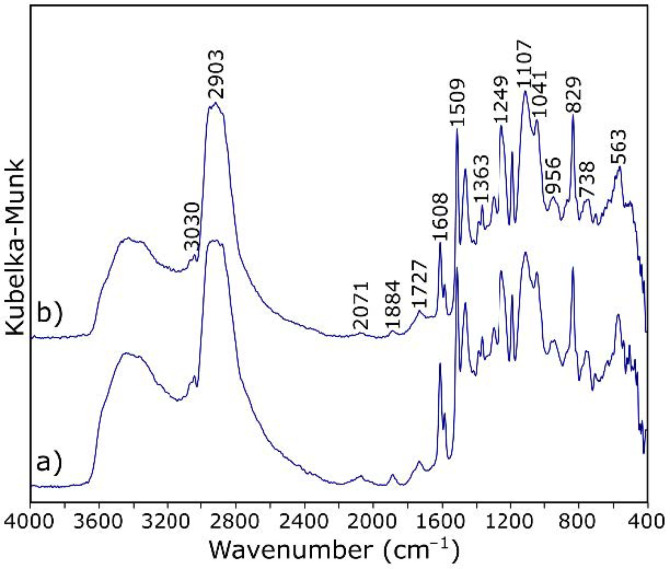
DRIFT spectra of (**a**) the carbon fiber reinforced polymers (CFRP) sample and (**b**) the CFRP sample with 0.3% GO addition.

**Figure 10:**
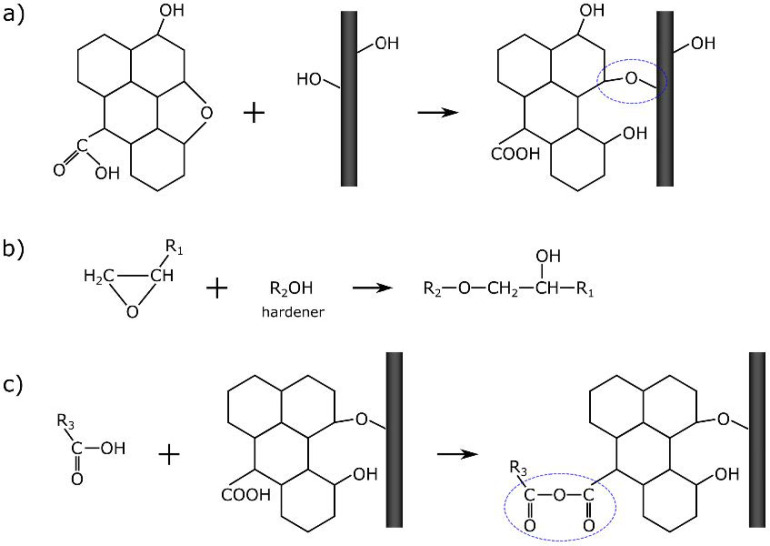
Proposed mechanism of GO influences on the CFRP composite: (**a**) GO to carbon fiber (CF) bonding [32]; (**b**) resin hardening reaction; and (**c**) proposed mechanism of combining fibers with resin.

Spectroscopic measurements of CFRP samples were also carried out. Figure 9 shows a comparison of the spectra of the composite without and with the addition of GO. Based on the analysis of their research results, Pathak et al. [32] formulated a thesis in which they proposed a way to connect GO with CF as schematically shown in Figure 10a. The CF used as a reinforcement possess hydroxyl functional groups, which attack the epoxy group of the GO; a chemical ether bond is formed as a result, and the surface development of carbon fiber increases. Therefore, the addition of GO to the composite should result in the formation of C–O–C (in another notation, >CH–O–CH<) bonds between GO and CF. In the case of a modified sample, the bands also from oxirane rings, and the bands related to OH groups involved in resin binding should disappear to a greater extent. While the number and sequence of the arrangement of the bands in the presented spectra (Figure 9) does not change, changes in their intensity ratios are noticeable. A decrease in the integral intensity of the bands at 3030 and 915 cm^−1^ (assigned to oxirane group; Table 1) is observed.

**Figure 11:**
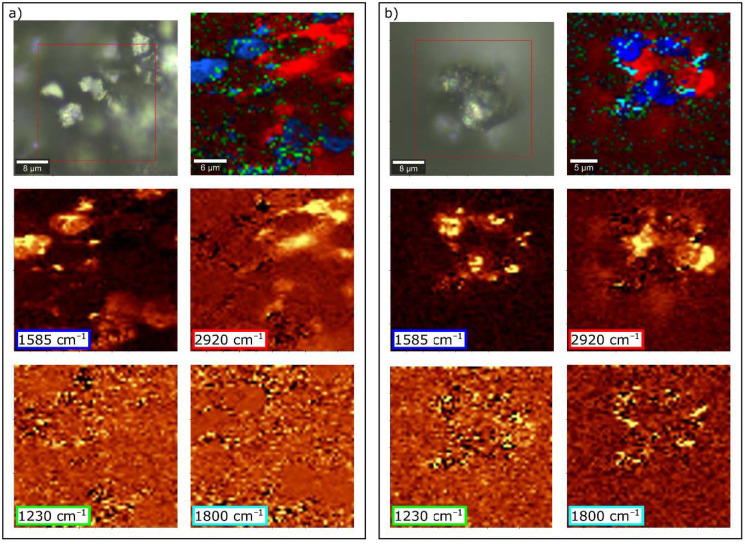
Raman images of (**a**) the reference CFRP composite and (**b**) the CFRP sample with 0.3% GO addition.

To confirm the nature of the bond formed between GO, CF and ER in composite, we have taken the Raman imagining of the CFRP composite (Figure 11). The spectra of individual components differ significantly from each other due to the fact that the analyzed materials do not have a homogeneous structure. Due to their similar chemical composition, the spectra of GO and CF are very similar, while ER shows a large number of peaks in its Raman spectrum (Figure 5). Moreover, there is a strong overlap between the Raman spectra of CF and ER. Therefore, the surface examination of samples did not bring the expected changes in the Raman spectra. Only imaging in the cross-section of the composite showed changes related to the vibrations of some bonds.

The hardening reaction of ER is performed by the epoxy group. This special molecular structure enables easy participation in addition reactions through opening the ring, and thus it polymerizes, as shown in Figure 10b. Bands corresponding to epoxide vibration are in the range of 1280–1230 cm^−1^ (vibration mode) and at 920, 740, and 643 cm^−1^ (deformation mode) [59]. The intensity of these peaks should be linearly dependent on the concentration of related groups. In this study, the breathing mode of the oxirane ring is at 1224 cm^−1^; however, there are no distinct changes related to this band. On the other hand, the bands attributed to the deformation of the oxirane ring are much weaker. It can be concluded that the addition of the GO does not affect the degree of polymerization of the resin. (The band at 1611 cm^−1^ should be inert to the polymerization reaction and not change its intensity.)

Pathak et al. [32,53] suggest that GO is binding with the epoxy polymer via H-bonding. As already mentioned, this is confirmed by the analysis of DRIFT spectra (Figure 9). In the Raman spectra, the only noticeable difference is observed in the intensity of the band at 1800 cm^−1^, as shown in Figure 11. The related vibrations are a symmetric C=O stretch of the acid anhydride functional group. The presence of this band within the CF/ER border zone suggests a relationship with the GO addition because, in the case of the reference sample, this band practically does not occur. A proposed mechanism for the formation of a bond between the resin and the system is shown in Figure 10c. The resulting acid anhydride group (marked loop in Figure 10c) may be a product of the reaction between the carboxyl groups of graphene oxide and the analogous hardener groups. This observation may be an additional explanation (to the best of our knowledge, so far unpublished) for better bonding of the fibers to the ER matrix in the case of samples with the addition of GO.

## 4. Conclusions

Analysis of the literature shows that the problem of improving the strength parameters of carbon fiber reinforced polymer composites is currently being investigated and focuses mainly on the improvement of adhesion at the fiber–matrix interface. The aim of this work was to investigate the addition of graphene oxide to the matrix of CFRP composites and the effect on their structural and strength properties. The results of the bending strength tests using the three-point static bending method showed that 0.3% of the weight is the optimal addition of GO. Such an addition caused an increase in bending stress by 18.4%, Young’s modulus by 35.5% and toughness by 18.8%. The greater addition of GO to the matrix resulted in a reduction of the mechanical properties of the laminate. It has been shown that by modifying the matrix, i.e., by appropriately doping the composite with nanoparticles, it is possible to strengthen the weakest element of this material, i.e., the CF/ER zone.

SEM microscopic observations confirmed that in the case of samples without GO, crack propagation took place along the interfacial boundaries, which are the weakest element of CFRP. The consequence was the phenomenon of pulling the fibers out of the matrix. In composites doped with GO, this effect is not observed. This proves good adhesion of the resin to the fiber, which contributes to the transfer of loads to the reinforcement.

The next step was to study the structure of composites using spectroscopic methods. Analysis of both IR and Raman spectra showed that the individual components interact with each other, and the material is not only a reinforcement physically enclosed in the matrix. By analyzing the spectra of the resin with and without the addition of GO, it was found that the vibration frequency of carboxyl group (–C=O) and hydroxyl group (–OH) shifts from 1736 to 1727 cm^−1^ and 3389 to 3356 cm^−1^, respectively, indicating the occurrence of hydrogen bonding between ER and GO. The addition of GO to the composite also results in the formation of C–O–C bonds between GO and CF, which increases the surface development of the fibers and the amount of available functional groups. It can be concluded that GO is an intermediate connection between the substrates of the laminate, connecting with the matrix by hydrogen bonding and with the reinforcement by chemical bonding. This conclusion was confirmed by Raman imaging of the samples’ fractures.

## Figures and Tables

**Table 1:** Characteristic bands of epoxy resin (ER) in the IR spectrum.

Band (cm^−1^)	Assignment [54,55]
3700–3100	O–H stretching
3030	stretching of C–H of the epoxy group (oxirane ring)
2965–2873	stretching C–H of CH_2_ and CH aromatic and aliphatic
1720	stretching –C=O of carboxyl group
1608, 1581, 1509	stretching C=C of aromatic rings
1249	stretching C–C of aromatic
1041	stretching C–O–C of ethers
915	stretching C–O of epoxy group (oxirane ring)
829	bending out of plane –CH in aromatic
738	rocking CH_2_

**Table 2:** Details of the quasi-static three-point bending test.

GO Addition (wt.%)	F_max_ (N)	ε_max_ (mm)	σ (MPa)	E (GPa)	Pp (J/m^2^)
0.0	93.1 ± 14.8	12.7 ± 1.3	654.6 ± 58.5	44.5 ± 4.3	50.4
0.1	86.7 ± 18.7	12.4 ± 1.3	678.2 ± 65.3	50.3 ± 7.5	51.9
0.2	80.0 ± 7.3	12.8 ± 0.7	678.7 ± 30.8	51.4 ± 2.1	53.6
0.3	84.1 ± 12.6	13.9 ± 1.0	774.9 ± 55.7	60.3 ± 9.2	59.9
0.4	86.0 ± 16.9	13.0 ± 1.6	724.4 ± 74.2	54.9 ± 9.4	54.9
0.5	72.4 ± 6.9	11.7 ± 0.7	623.2 ± 56.4	51.1 ± 2.7	43.4
1.0	73.2 ± 8.2	11.6 ± 0.7	609.9 ± 80.5	48.7 ± 13.5	43.3

## Data Availability

Data presented in this study are available on request from the corresponding author.
